# Case Report: Spinal proliferative meningitis with arachnoid diverticulum formation in a dog

**DOI:** 10.3389/fvets.2026.1864253

**Published:** 2026-08-27

**Authors:** Ivo Hajek, Kaspar Matiasek, Abby Caine, Viktor Palus

**Affiliations:** 1 Vetcentrum, AniCura Group, Prague, Czechia; 2 Section of Clinical and Comparative Pathology, LMU, Munich, Germany; 3 Granta Vet Specialists, Cambridgeshire, United Kingdom; 4 Neurovet, AniCura Group, Trenčín, Slovakia

**Keywords:** cysts, dogs, meningitis, MRI, spinal cord

## Abstract

This case report describes a 4-year-old Golden Retriever with chronic progressive motor impairment of pelvic limbs. A neurological examination revealed non-painful ambulatory paraparesis, and magnetic resonance imaging detected an eccentric cystic lesion that compressed the thoracic part of the spinal cord. The surgical approach consisted of spinal cord decompression and lesion excision, along with vertebral column stabilization. A neuropathological examination revealed dural and arachnoid hypertrophic calcifying changes with lymphoplasmacytic and neutrophilic infiltration. This is the first documented case of inflammatory meningoproliferative spinal cord disorder associated with arachnoid diverticulum formation in dogs.

## Introduction

Cystic abnormalities within the spinal cord (true cysts or pseudocysts/diverticula) are divided into several groups. Generally, we distinguish intramedullary (syringohydromyelia) and extramedullary lesions; the latter group includes meningeal and non-meningeal structures (1). Meningeal cysts cover extradural arachnoid diverticula without nerve root involvement (type I), extradural arachnoid diverticula with nerve root involvement (perineural—Tarlov cysts), and intradural cysts (2). Non-meningeal lesions include intraspinal (synovial and ganglion cysts) and discal cysts—hydrated nucleus pulposus extrusions (3, 4). Historically, causes in all cases are stated as congenital (anomalies), traumatic, or degenerative; in a few cases, an inflammatory association is anecdotally mentioned without any further description or visualization (1, 5). This case report aims to describe the diagnostic imaging and histopathological features of spinal hypertrophic meningitis with cystic compressive formation in a dog.

## Case description

A 4-year-old, 31 kg intact male Golden Retriever was referred for chronic (at least 6-month duration) progressive incoordination of the pelvic limbs. The dog was a pet; there was no history of trauma, and there were no similar symptoms in parents or littermates. Two years ago, there had been an episode of thoracic limb lameness and cervical pain, which raised a suspicion of steroid-responsive meningitis-arteritis (SRMA) without any additional diagnostic steps. After steroid treatment, the symptoms had disappeared. The patient also had a history of hip dysplasia, diarrhea, skin allergy, and otitis externa.

**Figure 1:**
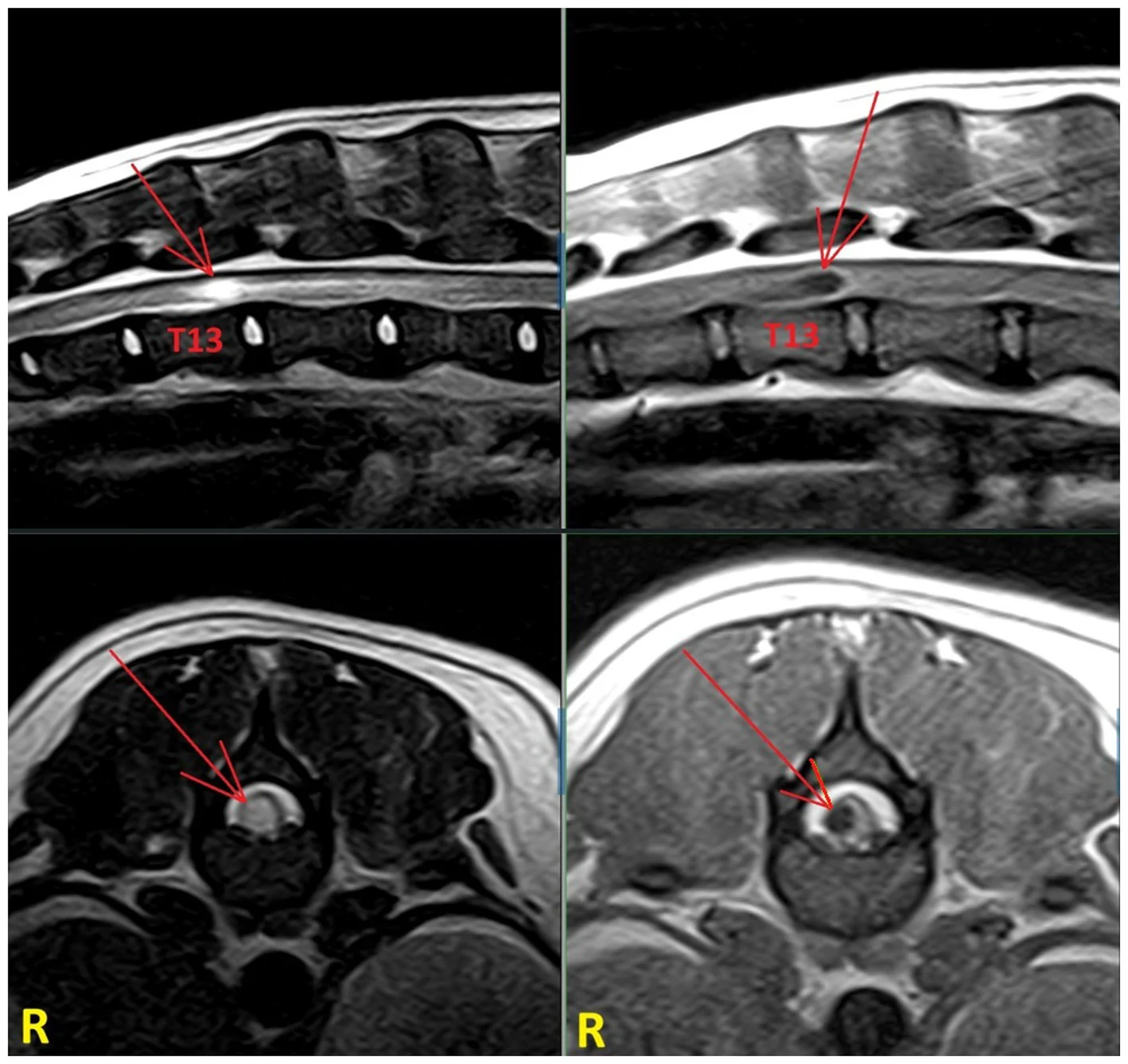
Upper left and upper right: T2W and T1W sagittal MRI images. Lower left and lower right: T2W and T1W + C transverse MRI images: A teardrop cystic structure in the spinal cord over the T13 vertebral body (red arrows).

**Figure 2:**
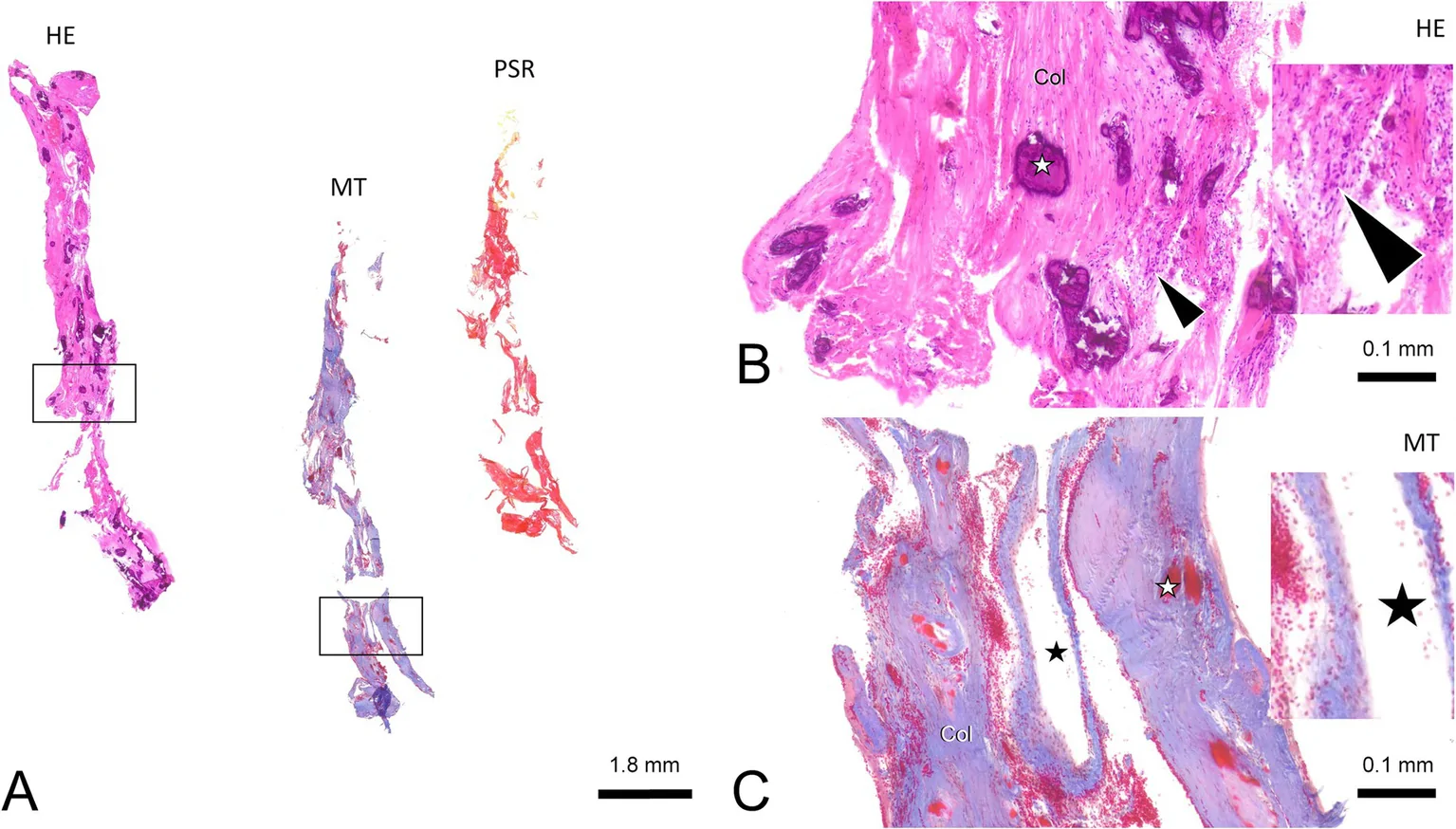
Histological presentation of the submitted biopsy at subgross **(A)** and microscopic **(B,C)** level: It mainly consists of dense pluricellular fibrocollagenous tissue. The bundles (**B,C**: Col) stain strongly blue (**A**: MT, **C**) or red (**A**: PSR) on special stains for collagen. Red reflection on polarized light on PSR specimens (not shown) proves them to consist mainly of mature collagen I. The tissue is peppered by islands of dystrophic calcification (**B,C**: white asterisks). Superficially, there are loose arrangements of arachnothelial cells mingling with mixed leukocytic infiltrates (arachnoiditis; **B**: black arrowhead). The membrane is multifocally interrupted by preformed clefts lined by flattened arachnothelium (**C**: black asterisk). Stains and abbreviations: Col: Collagen bundles; HE: Hematoxylin–eosin; MT: Masson’s trichrome; PSR: Picrosirius red. For magnification, see scale bars.

At the current presentation, a complete neurological examination revealed ambulatory, non-painful moderate paraparesis with noticeable posterior ataxia and decreased postural reactions in the pelvic limbs (proprioceptive deficits) with normal spinal reflexes in all four limbs. Cranial nerves, neck area, and thoracic limbs were normal. Neuroanatomic localization was consistent with a lesion localized between the third thoracic (T3) and the third lumbar (L3) spinal cord segments (T3-L3 myelopathy, upper motoneuron system for pelvic limbs). Differential diagnoses included inflammatory, traumatic, anomalous, neoplastic, and degenerative diseases. All parameters from the complete blood count (Idexx ProCyte Dx Hematology analyzer) and serum biochemical analysis (Idexx Catalyst Dx Biochemistry analyzer) were within normal limits, including the CRP level and SNAP 4Dx test. Diagnostic imaging included MRI of T3-L3 spinal cord segments. An MRI was performed under general anesthesia using a 0.3 T Esaote Vet-MR G-scan unit. Multiplanar T2-weighted (T2W), T1-weighted (T1W), and STIR sequences of the thoracolumbar region were acquired (T1W: TR: 520/680 ms, TE: 26 ms, FA: 90, ST: 4; T2W: TR: 2500/2600 ms, TE: 90 ms, FA: 90, ST 4; STIR: TR: 1600 ms, TE: 90 ms, FA: 40, ST: 4). Additional T1W images were obtained following the intravenous administration of a gadoteric acid-based contrast medium (Dotarem® 0.5 mmoL/mL, Guerbet, 0.1 mmol/kg). MRI images revealed an elongated, oval-to-teardrop-shaped space-occupying lesion (length 13 mm, height 5 mm) seen right dorso-laterally to the spinal cord at the level of the T13 vertebral body. It appeared intradural-extramedullary, with central intramedullary T2W hyperintensity extending two vertebral body lengths caudal to the lesion. The lesion was T1W hypointense and T2W/STIR hyperintense, with no enhancement of the lesion; however, some contrast enhancement was noted caudal and ventral to the lesion (Figure 1). The finding was considered an intradural cystic structure of unknown origin, with potential etiologies including an intradural congenital or acquired cyst, such as an arachnoid diverticulum, a neurenteric cyst, or cystic neoplasia. A decompressing surgery of the spinal cord was planned, based on the MRI examination. A standard hemilaminectomy was performed at the level of T13-L1, and due to potential predisposition to vertebral instability at the surgical site, vertebral stabilization of the T13-L1 segments was planned as a follow-up procedure. After the spinal canal was entered via T13-L1 hemilaminectomy, a durectomy was performed. The dura mater appeared macroscopically abnormal and calcified upon incision. A biopsy of the dura was taken and submitted for pathological examination. Beneath the dura, a cyst-like lesion was identified. The lesion macroscopically affected not only the subarachnoid space but also indented the spinal cord. The “wall” of the lesion was translucent, and after its excision, a fluid resembling cerebrospinal fluid (CSF) started leaking out. The cystic “wall” was then excised as much as was possible macroscopically and was submitted to the histopathological exam. A unilateral vertebral stabilization at the level of T13-L1 was performed using a 3.5 mm titanium SOP plate. The unilateral stabilization was deemed sufficient because the contralateral vertebral facet remained intact. A standard closure of the surgical site was performed. The tissue underwent routine processing for formalin-fixed paraffin-embedded tissue histology. The following stains were applied: hematoxylin–eosin, Giemsa stain, Masson’s trichrome, and picrosirius red. Histological slides were evaluated using bright-field (tissue composition and histoarchitectural and cytomorphological changes) and polarized light microscopy (orientation and composition of collagen fibers). The histological findings revealed the membrane to consist of thick, compact, quite regularly multilayered fibrocollagenous tissue (reminiscent of dura mater) with countless scattered, occasionally coalescing foci of dystrophic stromal mineralization. Polarized light microscopy reveals that the collagen consists of mature type I collagen. On the presumably inner side, the fibrocollagenous tissue shows multifocal loose arachnothelial proliferates with multifocal lymphoplasmacytic and neutrophilic infiltrates. The fibrocollagenous membrane furthermore appears multifocally retracted and clefted. These spaces were lined by flattened oligolayered arachnothelium (Figure 2). After receiving the histopathological results, prednisolone therapy (starting dose 2 mg/kg/day) was initiated with dosage reductions occurring at 2-week intervals; no additional treatment was given. Within 1 month post-surgery, the patient showed improvement in their ambulatory condition. Six months later, the patient was fully ambulatory with mild pelvic limb ataxia and was no longer receiving immunosuppressive therapy, instead undergoing long-term rehabilitation.

## Discussion

The meningeal histopathological changes in our patient corresponded to an inflammatory reaction in the dura mater and arachnoid membranes—meningitis is, in general, not a single disease but a syndrome associated with inflammatory meningeal irritation. Similar to humans, infectious and non-infectious causes are documented and all etiologic agents have been described in dogs (bacterial, viral, fungal, protozoal, rickettsial, and parasitic). Nevertheless, non-infectious syndromes of meningitis or meningoencephalomyelitis are seen more often in dogs and are considered to have an immunologic basis (6). The clinical signs are variable and depend on localization and process severity, with spinal pain and rigidity very common. Severe meningitis may lead to parenchymal inflammation of the brain or spinal cord, resulting in serious neurological deficits (7). In this patient, there was suspected cervical pain and thoracic limb lameness observed a few years prior to the current presentation, which was corticosteroid-responsive, with no further investigation. During the examination at our clinic for the current presentation, there was only motor function impairment of the pelvic limbs with no current evidence of spinal pain.

The mainstay of diagnostic work-up in neurological patients with suspected meningitis includes diagnostic imaging (MRI) and CSF examination. Our patient was paraparetic, non-painful, and MRI revealed cystic enlargement in the spinal cord at the level of the T13 vertebra; therefore, as meningitis was not prioritized as a differential diagnosis, a CSF tap was not performed. Due to the chronicity of clinical signs and massive spinal cord compression, the lesion was hypothesized to be causing progressive clinical signs as a slow-growing lesion. MRI indicated that the space-occupying lesion was cystic and was thought most likely to be intradural-extramedullary; however, an intramedullary localization was not completely ruled out. Generally, intradural-extramedullary cystic lesions are uncommon in T3-L3 localization (1). Acquired and congenital intradural arachnoid diverticula are described in this cord segment; however, the shape was not quite the typical appearance on sagittal sequences. A neurenteric cyst was included in the differential diagnosis for a cystic lesion near the cord. However, some neurenteric cysts may be isointense on T1W sequences (8), whereas others are described as T1W hypointense, as seen in the current case (9). Spinal cystic neoplasia is described, so was included as an unlikely consideration, given the intradural location (10). The dog underwent planned surgery under general anesthesia with a standard approach to the thoracic vertebrae and right hemilaminectomy, durectomy, and unilateral stabilization due to potential instability, as described in similar cases where it was stated to be sufficient (11–13). Histopathologic features in our case did not show any demarcation consistent with a true epithelium, but based on both clinical and histological features, an intradural arachnoid diverticulum (type III meningeal cyst) of the loculated and mineralized subtype, associated with multifocal lymphoplasmacytic and neutrophilic adhesive arachnoiditis, was diagnosed (1). Intradural meningeal cysts (arachnoid diverticula) are known by a variety of names (meningeal cysts, arachnoid, intra-arachnoid or subarachnoid cysts, leptomeningeal cysts or pseudocysts)—type I is more cyst-like because of being enclosed by a cystic wall, type III is not truly a cyst because of free communication with the subarachnoid space and a missing epithelial cell lining (1). In congenital forms, developmental abnormalities of arachnoid architecture are presumed with duplication or splitting of the arachnoid membrane during embryonic development, which may allow for its expansion throughout the patient’s life (2, 3, 14). Rare association with other congenital anomalies supports a genetic origin in some cases (15–17). Sometimes, a CSF flow disturbance as a functional one-way valve with fluctuations in CSF pressure is mentioned—this could result in the typical teardrop-shaped fluid accumulation (18, 19). The distribution of diverticula was so far mainly dorsal or dorsolateral (90%) and was restricted to the cranial cervical area and caudal thoracic aspect, with peak incidence at the C3 and T12 levels; clinical signs of patients correspond with neuroanatomical localization. Often described fecal incontinence was not observed in our patient (3). For those dogs with data available, intradural arachnoid diverticula have a predilection for the cervical region in large-breed dogs (85%), whereas 82% are found in the thoracolumbar region in small breeds (1, 20–22). Moreover, a recent study reported a high prevalence (82%) of syringomyelia associated with arachnoid diverticulum in the thoracolumbar region, which was not present in our case (23). Histopathologic evaluation submitted in dogs with arachnoid diverticula detected arachnoid defect and fibrosis of the subarachnoid membrane with meningeal cell proliferation; various pathologic changes may be seen within these structures, including hyperplasia and fibrosis (1, 14). In general, there is limited information on histopathology, and the data are largely anecdotal across several studies. In one case series, dural tissue was submitted for microscopic evaluation after durectomy—in two dogs, the dura and arachnoid layer were hypercellular with focal nodular aggregation, and the cellular inflammatory reaction was seen only in one dog. In other dogs from the series, the leptomeninges showed connective tissue proliferation with fibrosis, and adhesion of the pia to the arachnoid layer was normal. In another series were chondrodystrophic dogs with underlying intervertebral disc protrusions together with arachnoid diverticula or dogs after euthanasia which were historically managed with arachnoid diverticulum—in these cases, the histopathological examination detected connective tissue proliferation with fibrosis, spinal cord showed asymmetrical axonal degeneration and myelin loss with fibrous adhesion of the dorsal lamina to the dura and meningeal fibrosis with multifocal cystic cavitation of the dorsal funiculi (3, 14, 24, 25). None of the published cases described severe pachymeningitis or active arachnoiditis, as observed microscopically in our case (25). Arachnoiditis in human medicine was first reported already in 1907 (Krause). The arachnoid membrane and subarachnoid space do not contain any vessels but dura and pia are extremely vascular; therefore, the inflammatory response of arachnoid must originate from either the pia or dura—in our case, the inflammatory response and pathological changes of dura mater were also demonstrated (26, 27). Causes of arachnoiditis in humans include administration of contrast agents, infectious, traumatic, and hereditary impacts; and rare conditions such as arachnoiditis ossificans after spinal surgery as a potential cause of progressive myelopathy are described in humans (27–29). Spinal meningitis, especially adhesive arachnoiditis as a cause of cystic formation, is also a rare condition in human medicine, cited very sporadically. To the authors’ knowledge, it has never been reliably described in animals (30–33). Acquired arachnoid diverticula in dogs have been mentioned secondary to intervertebral disc disease or spinal cord trauma; however, an inflammatory spinal cord disease itself as a cause has not been described (1). Only one case has an acquired arachnoid diverticulum described as potentially associated with an inflammatory aetiology—in a Pug dog with concurrent intervertebral disc protrusion and positive *Toxoplasma gondii* antibodies in CSF and progressive clinical course after the decompressive surgery (1, 5, 14–16, 34–36) in which case it is not clear if the arachnoid diverticulum was congenital or acquired secondary to either the disc or inflammation.

According to the MRI features and histological findings in our patient, we postulate that this case was an arachnoid diverticulum associated with an inflammatory process—chronic SRMA can be the differential diagnosis since the Golden Retriever is a predisposed breed but there is no evidence in the literature linking SRMA and the development of arachnoid diverticula (37). The limitations of the report are the lack of macroscopic intraoperative visualization, the absence of CSF evaluation at the current or previous timepoints, and the use of superior MRI sequences such as HASTE or 3D-CISS (38, 39). To the authors ´ knowledge, this could be the first report of spinal arachnoid diverticulum formation associated with arachnoiditis, which may relate to a previous inflammatory process, analogous to the pathogenesis cited in human medical literature.

## Data Availability

The original contributions presented in the study are included in the article/supplementary material, further inquiries can be directed to the corresponding author.
